# Cerebral oxygenation and skeletal muscle responses to time trial, maximal oxygen uptake and time to exhaustion exercise

**DOI:** 10.1113/EP093170

**Published:** 2026-05-15

**Authors:** Caroline V. Robertson, Nicole T. Vargas, Tegan Hartmann, Frank E. Marino

**Affiliations:** ^1^ Queensland Centre for Mental Health Research University of Queensland Wacol Australia; ^2^ School of Allied Health, Exercise and Sports Sciences Bathurst, New South Wales Australia; ^3^ School of Medicine and Psychology, College of Health and Medicine The Australian National University Canberra, Australian Capital Territory Australia; ^4^ School of Rural Medicine, Leeds Pde Charles Sturt University Orange, New South Wales Australia

**Keywords:** cerebral oxygenation, EMG, exercise intensity, NIRS, time to exhaustion

## Abstract

This study investigated cerebral and neuromuscular responses to three exercise models: time trial (TT), maximal oxygen uptake (${\dot{V}}_{O_{2}\max}$) and time to exhaustion (TTE). Fourteen participants completed the tests in the following order: ${\dot{V}}_{O_{2}\max}$, TT and TTE. To compare responses across different intensities, TT and ${\dot{V}}_{O_{2}\max}$ trials were matched for total work (kJ), and the average TT power output was used to set intensity for TTE. Throughout exercise, near‐infrared spectroscopy from the prefrontal cortex and electromyography from the vastus lateralis and rectus femoris were recorded. Ratings of perceived exertion and the feeling scale were collected post‐exercise. Total work was similar across trials. Tissue oxygenation index (TOI) was significantly lower during TT (*P *= 0.0308) and TTE (*P *= 0.0400) than ${\dot{V}}_{O_{2}\max}$. Deoxyhaemoglobin increased more during TTE (*P *< 0.0001), and oxyhaemoglobin was higher during TTE from 60% to 100% of test completion (*P *= 0.0040–0.0001). Participants rated ${\dot{V}}_{O_{2}\max}$ as more pleasant (6.7 ± 2.9) than TT (5.9 ± 3.0, *P *= 0.0412) and TTE (4.9 ± 2.3; *P *= 0.0124). Despite equivalent work, each exercise model elicited distinct neurophysiological profiles and distinct temporal patterns of prefrontal oxygenation and muscle activation. TT and TTE showed earlier reductions in TOI than ${\dot{V}}_{O_{2}\max}$, but end‐exercise brain oxygenation was similarly reduced across paradigms, while ${\dot{V}}_{O_{2}\max}$ was perceived as more pleasant. These findings suggest that exercise modality alters the trajectory, rather than the endpoint, of prefrontal oxygenation/perfusion responses.

## Abstract (highlights)

- **What is the central question of this study?**
  Do common exercise tests (${\dot{V}}_{O_{2}\max}$, time trial, time‐to‐exhaustion) produce different time courses of prefrontal cortex perfusion and oxygenation when tracked with total haemoglobin (tHb) and haemoglobin difference (Hbdiff) and tissue oxygenation index (TOI)?
- **What is the main finding and its importance?**
  Based on combined NIRS indices (tHb, Hbdiff), it appears that cerebral perfusion built late during incremental ${\dot{V}}_{O_{2}\max}$ test, mid‐to‐late in the self‐paced time trial, and gradually from early to late in the time to exhaustion test, while extraction shifts were modest and TOI fell progressively. Even though the three different exercise tests showed different prefrontal cortex perfusion patterns, they all ended with a similar drop in brain oxygenation; the exercise protocols changed the time course but not the endpoint.

## 1 INTRODUCTION

The maximal oxygen uptake (${\dot{V}}_{O_{2}\max}$), time to exhaustion (TTE) and the time trial (TT) tests are the three most widely utilised tests to evaluate and understand the limitations to exercise capacity and to examine the effect of interventions on endurance. The ${\dot{V}}_{O_{2}\max}$ and TTE tests are both open‐ended tests, whereby the participant continues to exercise for as long as possible until volitional exhaustion. The ${\dot{V}}_{O_{2}\max}$ is traditionally incremental in power output (PO), whereas the TTE is a constant load (speed or power) and continues until the required work rate can no longer be maintained. The TT differs in that it typically involves exercise of a known duration or distance, to be completed in the fastest possible time and has a known endpoint. The TT has gained popularity as it is thought to be more ecologically valid (Currell & Jeukendrup, 2008) and more reliable (Jeukendrup et al., 1996; Hinckson & Hopkins, 2005) than a TTE, although this view has been challenged.

Comparisons between these tests (Neary et al., 2002) have added to the debate surrounding mechanisms of exercise regulation and termination initially described as teleoanticipation (Ulmer, 1996) and subsequently extended by the Central Governor (Noakes et al., 2001; St Clair Gibson & Noakes, 2004) and anticipatory regulation models (Marino, 2004). The TT allows adoption of a pacing strategy which means that, compared to a sustained effort at the same average PO, the physiological strain is less (Lander et al., 2009). This manipulation of physiological strain suggests the presence of regulation between the working organs and the central nervous system (CNS) (St Clair Gibson & Noakes, 2004) to ensure that demand is not so high that a meaningful slowdown can occur and yet the event is completed as fast as possible (Foster et al., 2003; St Clair Gibson et al., 2018). This pacing is thought to be regulated by afferent feedback which alters motor unit recruitment (Amann & Dempsey, 2008; Amann et al., 2006, 2007) by the CNS (Amann, 2012) and is thought to be a product of the interaction between feedback and feedforward mechanisms of exercise limitation (Hureau et al., 2018). However, it is also postulated that an anticipatory mechanism provides feed‐forward capability which, regardless of the level of peripheral fatigue at the end of exercise, can produce additional recruitment from previously quiescent motor units activated during the endspurt (ES) (Ansley et al., 2004) thought to be indicative of the self‐regulatory characteristics of this event. As such, a greater understanding of the cerebral correlates of the ES could increase our understanding of the self‐regulatory nature of this event in comparison to the TTE and ${\dot{V}}_{O_{2}\max}$ tests.

In comparison to a TT, the end point of an incremental test to exhaustion (${\dot{V}}_{O_{2}\max}$) has been described previously as potentially linked to alterations in cerebral metabolism (Rasmussen et al., 2010; Robertson & Marino, 2015) resulting from the hyperventilatory response to increasingly altered arterial carbon dioxide tension. It has also been previously proposed that the ${\dot{V}}_{O_{2}\max}$ test may not be limited by the cardiovascular system (Noakes, 2008; Noakes & Marino, 2009). Therefore, the cardiovascular system may not be working maximally at the end point of an incremental test, which may be due to alterations in cerebral homeostasis. Exhaustive exercise has previously been shown to limit cerebral blood flow (Linkis et al., 1995), decrease cerebral oxygenation (Rupp et al., 2008) and limit mitochondrial tension (Rasmussen et al., 2010), and alter the electroencephalography responses (Robertson & Marino, 2015). We hypothesised that the incremental ${\dot{V}}_{O_{2}\max}$ test would evoke the greatest skeletal muscle deoxygenation versus the TT and TTE protocols, in line with deoxygenation scaling with work rate and recruitment (Perrey et al., 2024; Rooks et al., 2010). At the same time, protocol effects near maximal effort such as a higher ${\dot{V}}_{O_{2}\max}$ in a decremental versus incremental test (Beltrami et al., 2012, 2022), caution that near‐maximal oxygenation may not increase monotonically with external workload.

We have previously proposed a role for the prefrontal cortex (PFC) (in combination with other brain areas) in exercise tolerance, regulation and termination in conjunction with internal and external exercise stimuli (Robertson & Marino, 2016). Since then, further theories involving the PFC and exercise performance (Hyland‐Monks et al., 2018; Lutz, 2018; McMorris et al., 2018) and the role of sensory‐discriminatory, affective‐motivational and cognitive‐evaluative processes have been proposed (Venhorst et al., 2018). These theories posit that depending on the nature of the exercise model being performed, the brain will respond accordingly to the multiple challenges the body faces in order to continue exercising. Data supporting the role of the PFC in exercise performance have also been published (Angius et al., 2019; Santos‐Concejero et al., 2017, 2017). Therefore, the aim of this study was to compare the neurophysiological responses to these three tests. Our hypothesis was that they would display different neurophysiological characteristics due to different cerebral requirements and physiological nature of the tests.

## 2 METHODS

### 2.1 Participants and ethical approval

Fourteen well trained cyclists (11 male and 3 female) from the local cycling and triathlon clubs volunteered for this study. Their weekly distance (km) and years of training were 251 ± 95 km and 8 ± 5 years, respectively. Their age, height, weight, ${\dot{V}}_{O_{2}\max}$, absolute peak power, peak power relative to mass, and percentage body fat (mean ± SD) were 38 ± 12 years, 176.8 ± 8.4 cm, 77.6 ± 13 kg, 4.6 ± 1.1 L min^−1^, 394 ± 70.1 W, 5.1 ± 0.7 W kg^−1^ and 13.5 ± 6.5%. After being informed of the risks associated with the experiment, each participant signed a letter of consent. Participants were not on any medications and had no symptoms of cardiovascular, neurological or respiratory diseases. All participants were carrying out endurance training at the time of the study and were not participating in any weight training. All females were tested during the follicular phase of their menstrual cycle (Maher et al., 2010). The study was approved by the Research and Ethics Committee of the University (Protocol number 2013/022) and conformed to standards set by the latest revision of the *Declaration of Helsinki*.

### 2.2 Procedure

**FIGURE 1:**
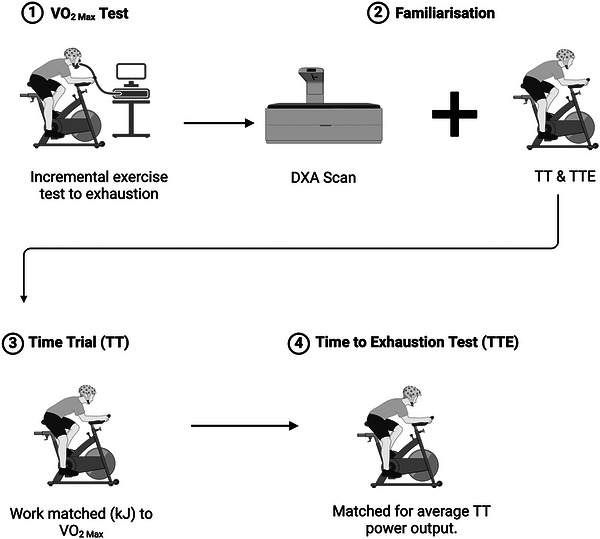
Study design schematic with four visits, a ${\dot{V}}_{O_{2}\max}$ test, familiarisation, time trial (TT) and time to exhaustion (TTE).

Participants attended the laboratory on four separate occasions (1 × familiarisation, 3 × exercise tests; see Figure 1 for experimental design). All test sessions were scheduled to take place at the same time of day with controlled temperature conditions (20 ± °C, 40% relative humidity). Prior to each visit, participants were asked to refrain from any exercise for at least 24 h prior to testing and to undertake only easy training from 36 h prior to each visit. Each visit was a minimum of 48 h apart and all test sessions were completed within a maximum time frame of 14 days. All participants maintained a regular diet during the study period and were asked to refrain from alcohol ingestion for at least 24 h prior to testing and caffeine 6 h before, which was confirmed by a self‐reported food diary.

### 2.3 Experimental overview

All test sessions were completed on a cycle ergometer (Excalibur Sport, Lode, Groningen, the Netherlands), the measurements for which were as close to their own bike as possible and were recorded and replicated for each trial. Tests were performed in a quiet room with no internal or external interruptions and no verbal encouragement was provided during any tests. All participants completed the tests in the following order: ${\dot{V}}_{O_{2}\max}$ test, familiarisation, TT and TTE. The order of the trials was completed in this way to allow both the ${\dot{V}}_{O_{2}\max}$ and the TT to be work matched. This was deemed the best way to allow for physiological comparisons between the two tests due to the changes in intensity within the ${\dot{V}}_{O_{2}\max}$ test. To be able to work match the two tests, an amount of work from the ${\dot{V}}_{O_{2}\max}$ test was required before being able to complete the TT. To be able to match the TT and the TTE, the best way was deemed to match the efforts for intensity, so that the level of afferent feedback between each trial was similar. Potentially a PO could have been set for the TTE based on a percentage of ${\dot{V}}_{O_{2}\max}$, but this would have yielded the same intensity within participants but not between tests, making the TT and TTE less comparable. The core aim of this study was to examine differences between tests.

Participants were given no information about the purpose of the study, to ensure they could not manipulate the tests. To ensure minimal external input that could alter neurophysiological responses, the following criteria were in place during testing. During the ${\dot{V}}_{O_{2}\max}$ test and TTE participants were given no performance feedback, including cadence while looking only at a blank wall. During the TT the only feedback they received was total work (kJ) completed. Further, no routine measurements were taken during exercise and no music or fluids were permitted during the trials.

Prior to each exercise test, participants were instrumented with two near infrared spectroscopy (NIRS) probes and two electromyography (EMG) electrodes (described subsequently) and a heart rate monitor. Resting measurements for NIRS were collected for 1 min with eyes open (EO). During this time participants remained as still as possible and were asked to look straight ahead and think of nothing. Following resting measures, participants undertook a standardised warm up of 100 W for 5 min. All data were collected with Lode Ergometry Manager software version 10 (Lode).

### 2.4 Maximal oxygen consumption test (${\dot{V}}_{O_{2}\max}$): visit 1

Participants were fitted with a facemask (Hans Rudolph, Shawnee, KS, USA) connected to a rapid response gas analyser (AEI Technologies, Pittsburgh, PA, USA) for the measurement of pulmonary gas exchange. A 1‐min resting sample was collected before the initiation of the test. Cyclists performed a cycle ramp test to exhaustion starting at 100 W and consisting of a ramp increase in PO of 25–30 W min^−1^ (depending on individual ability) until volitional exhaustion. Participants were asked to maintain their preferred cadence (all participants selected between 80 and 100 rpm) for as long as possible. The test was terminated voluntarily by each participant when they could no longer sustain the required PO whilst staying seated in the saddle or by the investigator when the pedalling rate dropped below 40 rpm. The total amount of work (kJ) completed during the test was recorded and the TT was matched for this amount of work.

### 2.5 Familiarisation: visit 2

#### 2.5.1 Dual X‐ray absorptiometry scan

Body fat percentage, total lean mass and lean mass of the lower limbs were assessed by a whole‐body dual X‐ray absorptiometry (DXA) scan. DXA equipment was calibrated using a quality assurance calibration standard and a quality control ‘phantom’ to determine precision for bone mineral density, fat and lean mass. Scanning resolution was set at 6.5 × 13.0 mm and scanning speed was set at 130 mm/s (Illuminatus DXA User Interface Software Version 4.2.0). Participants were scanned in a supine position. The regional analysis of the whole‐body scan was used to calculate lower limb lean mass (kg) (legs and gluteal area) as reported previously (Perez‐Gomez et al., 2008).

#### 2.5.2 Exercise bouts

During familiarisation participants performed a TT which was set at the total amount of work completed in the ${\dot{V}}_{O_{2}\max}$ test. The TT was completed in linear mode, whereby the ergometer acts as a mechanically braked system which increases in pedalling rate to allow an increase in work rate according to the following formula:

$$W=L^{\;.}\;{\left(\mathrm{RPM}\right)}^{2}$$

where *RPM* is pedalling rate and *L* is a constant linear factor. This alpha factor was chosen in such a way to allow for the average cadence achieved in the ${\dot{V}}_{O_{2}\max}$ test at a PO similar to 85% of respiratory compensation point (RCP). Participants were requested to complete the set amount of work as quickly as possible as in a race situation. This method has been used previously in work using TT performance (Jeukendrup et al., 1996). Following familiarisation, participants could alter the alpha linear factor for the actual TT. After a 30 min rest, participants then completed a TTE with the load set at the average PO achieved during the TT. The TTE was completed in hyperbolic mode whereby a constant work rate can be imposed and will remain constant, independent of the participant's pedalling rate. The test was terminated once the participant could no longer sustain the power required or when the pedalling rate dropped below 40 rpm.

#### 2.5.3 Time trial (TT): visit 3; and time to exhaustion (TTE): visit 4

On their third visit to the laboratory, participants completed a TT which consisted of the same amount of work (kJ) as that completed in the ${\dot{V}}_{O_{2}\max}$ test with the test carried out as per the familiarisation trial. The only feedback was total kJ completed which was viewed on a computer screen and provided by the Lode Ergometry Manager (LEM) computer software. The average PO achieved during the TT provided the load required for the TTE. Participants were not informed of the PO prior to the trial. On the fourth visit, participants completed TTE during which they were given no performance feedback; the only instructions given to the participants were to keep going for as long as they could. The trials were matched for intensity (average PO) to make them as comparable as possible.

### 2.6 Measurements

Pulmonary gas exchange was measured breath‐by‐breath throughout the ${\dot{V}}_{O_{2}\max}$ tests using a custom‐designed expired gas analysis metabolic system. Expired gas analysis was undertaken using a 2‐litre mixing bag placed on the expired port of the mouthpiece, and mixed expired air was sampled continuously and pumped to rapid‐response gas analysers (AEI Technologies). Ventilation was measured by a flow turbine (UVM; VacuMed, Ventura, CA, USA) connected to the inspired side of the mouthpiece. This method has been previously described and validated (Robergs, 2014; Robergs et al., 2010). All data were acquired using custom‐developed software (LabVIEW; National Instruments, Austin, TX, USA) and commercial electronic acquisition devices (National Instruments). The volume transducer was calibrated before each test with a 3‐l calibration syringe (Hans Rudolph), and the analysers were calibrated with gases of known concentration: 100% N_2_, room air and medically certified calibration gas of 16% O_2_, 5% CO_2_.

### 2.7 Ventilatory parameters

Breath‐by‐breath gas exchange data from all ${\dot{V}}_{O_{2}\max}$ tests were transferred to a spreadsheet program (Microsoft Excel) for further analysis. All ${\dot{V}}_{O_{2}}$ data were time averaged over eight breaths. The ${\dot{V}}_{O_{2}\max}$ was determined by the highest serial average of eight breaths, as has been performed previously (Robertson & Marino, 2015). Ventilatory thresholds were determined using previously defined methods utilising increases in the ventilatory equivalents (Amann et al., 2006; Caiozzo et al., 1982). The VT was determined by an increase in ${\dot{V}}_{E}/{\dot{V}}_{O_{2}}$ and the RCP by the second increase in ${\dot{V}}_{E}/{\dot{V}}_{O_{2}}$ with a concomitant increase in ${\dot{V}}_{E}/{\dot{V}}_{CO_{2}}$ (Bhambhani et al., 2007; Caiozzo et al., 1982; Davis, 1985). Expired‐gas and ventilatory data were not collected during either TT or TTE, so ${\dot{V}}_{E}$, ${\dot{V}}_{O_{2}}$ and RCP are reported for ${\dot{V}}_{O_{2}\max}$ only.

### 2.8 Electromyography

Prior to exercising, participants were prepared for the placement of surface electromyographic (EMG) electrodes (Delsys, Boston, MA, USA) on the vastus lateralis (VL) and rectus femoris (RF) on the right leg. Differential surface electrodes (Delsys) were placed over the belly of the muscle parallel to the muscle fibres. Prior to electrode placement, the skin over the muscle was shaved and the outer layer of epidermal cells was abraded, and oil and dirt cleaned from the skin with an alcohol swab. The EMG electrodes consisted of a parallel bar configuration with an inter‐electrode distance of 10 mm. These were adhered to the skin and wires were threaded under each participant's shorts to reduce movement artefact. The position of each electrode was measured from the proximal border of the patella and noted to ensure reliability of electrode placement across visits. A gel adhesive reference electrode was placed on an electrically neutral and mechanically stable site. The electrodes were connected via an insulated cable to the signal acquisition and amplification apparatus (Bagnoli‐8, Delsys; DAQ A/D card, National Instruments) and acquired signals were saved to custom acquisition software (LabVIEW).

### 2.9 Near‐infrared spectroscopy

Two near‐infrared spectrometer (NIRS) probes (NIRO‐200, Hamamatsu Photonics, Hamamatsu City, Japan) were placed over the left and right prefrontal lobes between FP1 and F3 (left) and FP2 and F4 (right) (Billaut et al., 2010). The NIRS probes were adhered to the skin with each probe pair placed in a black plastic probe holder, with predefined inter‐optode spacing of 4 cm. The larger inter‐optode distance was chosen to provide a greater maximum depth measured (Okada et al., 1995). The emission and detection probes were placed in opposite directions for each channel and the detection probes placed next to each other when on the forehead. The probes were housed in black rubber holders and attached to the skin with double sided adhesive discs. The probe holders were also secured in place by a black headband to reduce any extraneous light infiltration and to prevent any change in their position during exercise. Data were sampled at 1 Hz. Given that we did not have an a priori lateralisation hypothesis, left and right traces were averaged at each time point to yield an overall PFC signal for OHb, HHb, tHb (OHb + HHb) and Hbdiff (OHb − HHb). This approach increases precision and avoids multiplicity from redundant channels when balanced. As such, all NIRS results reported refer to the overall PFC signal.

### 2.10 Subjective measures

At the immediate completion of exercise, participants were asked to provide a score from two scales: the rating of perceived exertion (RPE) (Borg, 1990) and the feeling scale (FS). The FS (Hardy & Rejeski, 1989) was used as a measure of affect immediately post exercise. The FS measures basic affect (pleasure–displeasure) (Ekkekakis & Acevedo, 2006). The scale is an 11‐point scale ranging from −5 to +5 with verbal anchors at odd integers and at the zero point: −5 very bad, −3 bad, −1 fairly bad, 0 neutral, 1 fairly good, 3 good, 5 very good. Affect plays a role in how well exercise intensity is tolerated and may impact the specific response chosen to accommodate uncomfortable sensations (Robertson & Marino, 2016; Tempest & Parfitt, 2016).

### 2.11 Data analysis

#### 2.11.1 EMG analysis

EMG signals were imported into a custom‐made analysis program for processing (LabVIEW). EMG signals were first processed to detect baseline signals and bandpass filtered at 20 and 450 Hz to remove noise. The filtered data were then processed on a contraction‐by‐contraction basis based on a detectable change from baseline. The contraction signals were saved and then subsequently processed for root mean square data and duration of contraction. In addition, the contraction signals were processed to determine mean and median frequency data derived from power spectral density and a Hanning window. Data from each test were then normalised to the maximal data achieved during the ${\dot{V}}_{O_{2}\max}$ test.

#### 2.11.2 NIRS analysis

Changes in concentration of oxyhaemoglobin ($\Delta O_{2}\mathrm{Hb}$) and de‐oxyhaemoglobin (ΔHHb) were calculated using the modified Beer–Lambert law, based on changes in light attenuation at three wavelengths and the adult forehead differential path length factor of 5.93 (Van der Zee et al., 1992). Tissue oxygenation index (TOI) and normalised tissue haemoglobin index (nTHI) were measured by the spatially resolved spectroscopy method. The TOI is a percentage ratio of O_2_Hb to total haemoglobin (THb) and represents the oxygen saturation of tissue haemoglobin. The nTHI is the sum of O_2_Hb and HHb expressed as a ratio, which represents the relative concentration of total tissue haemoglobin and is, therefore, an indicative measure of regional blood volume (Van Beekvelt et al., 2001). NIRS was referenced to the first stable in‐task epoch (1% of normalised test duration) rather than seated rest to minimise posture and transition artefacts and potential arbitrary baseline drift inherent to continuous‐wave NIRS. This ‘freshest on‐task’ anchor provides a common physiological context across paradigms (all moving at low workload) and reduces between‐session variability from anticipatory changes and sensor settling.

Following testing, data were imported into an Excel spreadsheet for further analysis. Values were normalised to the first percent of exercise and calculated as absolute change. Absolute change was used instead of percentage change to avoid high values (>1000% change) seen in some NIRS variables when percent change is calculated. All data for each NIRS parameter and EMG were normalised to 100 points (1 point per percentage of exercise duration). Data were then also distributed further into ‘bins’ for every 10% of the test completed for further analysis.

#### 2.11.3 Determination of the endspurt

The ES has not yet been determined using high resolution data. Previous research has used a visual analysis approach over longer periods of time (Catalano, 1973, 1974) or distance (Lima‐Silva et al., 2010; Micklewright et al., 2010). This is likely due to the power fluctuations that occur throughout the TT which make this type of determination difficult (Tucker et al., 2006). Due to the continued interest and identification of the ES, it was considered important to begin a more in‐depth analysis of this phenomenon. A set of guidelines were, therefore, constructed to enable determination of the ES in each TT as follows:

1. Power data were averaged over 5 s. This equated to approximately 200 m which has been used previously to represent average PO during cycling TT (Tucker et al., 2006).
2. Data were transferred into a software programme (LabVIEW) for further processing;
3. The time–power curve was plotted and visually analysed.
4. The lowest point (*x*), prior to an increase in PO which continued towards the end of exercise, was identified.
5. Any negative changes from this point on were required to be greater than a 2.5% increase from the initial low point (*x*) to the lowest point identified (*y*) within the drop in power. Therefore, the change from point *x* to *y* was required to be greater than 2.5%. A 2.5% change was chosen based on previous research examining the ES and the percentage change required for an increase in the variable of interest to be significant (Catalano, 1973, 1974). Based on these data to ensure that the increase between the initial start point of the ES and the lowest point following a decrease was still a significant increase, the margin of 2.5% was chosen.

### 2.12 Statistical analysis

Normality of all data were tested by using the Shapiro–Wilk test. Perceptual and psychological data were then analysed using a one‐way repeated measures ANOVA with a Bonferoni *post hoc* correction factor. Physiological data (PO, NIRS, EMG and HR) were analysed using a two‐way general linear model repeated measures ANOVA for the factors test (${\dot{V}}_{O_{2}\max}$, TT, TTE) and time (1%, 10%, 20%, 30%, 40%, 50%, 60%, 70%, 80%, 90%, 100%) where applicable. Following a significant *F*‐test, pair‐wise differences were identified using a Bonferroni *post hoc* correction factor. Following a significant omnibus test, Bonferroni‐adjusted pair‐wise comparisons were used to examine within‐test changes and between‐test differences across the normalised 10% time bins. Primary interpretation focused on the overall paradigm × time trajectory, with particular attention to late‐task behaviour (50%, 90% and end‐exercise/end‐spurt). *Post hoc* single‐time‐point contrasts were not carried out, nor were correlation or stepwise regression screens to avoid selective inference and instability. Workload equivalence across paradigms was neither intended nor achievable within‐subject; thus, between‐paradigm comparisons were based on the full trajectory rather than arbitrary points. For cerebral NIRS we also derived tHb (OHb + HHb) and Hbdiff (OHb − HHb) at each 10% bin (values referenced to the 1% on‐task epoch) and analysed them in the same test × time repeated‐measures ANOVA.

## 3 RESULTS

### 3.1 Maximal oxygen uptake, time trial and time to exhaustion

Tests were designed to be matched for work completed (kJ; ${\dot{V}}_{O_{2}\max}$ and TT) and intensity (average PO; TT and TTE). Total work done and total amount of time completed for each test are shown in Table 1. Although TTE showed a numerically higher and more variable total work, this was not statistically different. Total work did not differ between tests (Table 1). Total time was lower in TT than in ${\dot{V}}_{O_{2}\max}$ (*P* < 0.0001), whereas ${\dot{V}}_{O_{2}\max}$ and TTE did not differ. For reference, the average intensity of the TT, and therefore the load for the TTE, was 81.8 ± 5.8% of peak *W* obtained in the ${\dot{V}}_{O_{2}\max}$ test. For reference, the average intensity of the TT and therefore load for the TTE was 81.8 ± 5.8% peak *W* obtained in the ${\dot{V}}_{O_{2}\max}$ test.

**TABLE 1:** Comparison of physiological and psychological parameters between each exercise model.

Test	Total work done (kJ)	Total time completed (s)	Average HR (bpm)	Max HR (bpm)	RPE	FS
${\dot{V}}_{O_{2}\max}$	150.8 ± 54.6	593 ± 134.8	143.7 ± 8.2*^b^*	175.1 ± 7.5	17.7 ± 1.3	6.7 ± 2.8*^a^*
TT	150.8 ± 54.6	459.9 ± 107*	160.1 ± 9.7	173.7 ± 9.2	17.9 ± 1.6	5.8 ± 3
TTE	194.1 ± 135.1	597.9 ± 407.6	159.6 ± 9.7	172 ± 10.9	18 ± 1.7	4.9 ± 2.3

*Note*: TT vs. ${\dot{V}}_{O_{2}\max}$ *P < 0.0001 and TTE *^b^P *< 0.0001. ${\dot{V}}_{O_{2}\max}$ vs. TT *^a^P *= 0.0412 and TTE *P *= 0.0124 (*n* = 14). FS, feeling scale; HR, heart rate in beats per minute (bpm); RPE, rating of perceived exertion; TT, time trial; TTE, time to exhaustion; ${\dot{V}}_{O_{2}\max}$, maximum oxygen uptake test.

### 3.2 Power output

**FIGURE 2:**
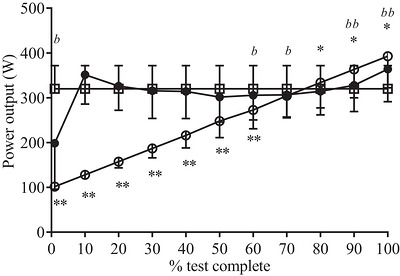
Changes in power output (W) across ${\dot{V}}_{O_{2}\max}$ (¡), TT (l) and TTE (o) tests. Values are means ± SD for 14 participants, with each participant contributing repeated measures across normalised % test‐completion bins. ${\dot{V}}_{O_{2}\max}$ was lower than both TT and TTE from 1% through to 60% (**P = 0.0001) and higher than the TT at 80% (*P = 0.0283), 90% (*P = 0.0164) and 100% (*P = 0.0244) and higher than TTE at 90% and 100% (*^bb^P = *0.0001). TTE was higher than TT at 1% (*^b^* *P *< 0.0001), 60% (^*b*^ *P *= 0.025) and 70% (*^b^P *= 0.0194) and lower 90% and 100% (*^bb^P *= 0.0046). TT, time trial; TTE, time to exhaustion; ${\dot{V}}_{O_{2}\max}$, maximal oxygen uptake test.

There was a main effect for test and time and a test × time interaction for PO. Average PO from the ${\dot{V}}_{O_{2}\max}$ test was significantly lower than from the TT and TTE test (245.9 ± 97.01 vs. 311.4 ± 42.2 vs. 320 ± 52, respectively) (*P* = 0.0001), whilst being significantly higher at 80% (*P* = 0.0283), 90% (*P* = 0.0164) and 100% (*P* = 0.0244) of the TT and 90% and 100% of the TTE (*P* = 0.0001) (see Figure 2).

### 3.3 EMG

**FIGURE 3:**
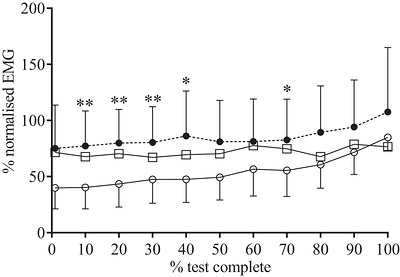
Changes in normalised electromyography (EMG) response during the ${\dot{V}}_{O_{2}\max}$ (¡), TT (l) and TTE (o) tests. Values are means ± SD for 11 participants, with each participant contributing repeated measures across normalised percentage test‐completion bins. TT was significantly higher than ${\dot{V}}_{O_{2}\max}$ at 10%, 20% and 30% (**P = 0.0054, 0.0002, 0.0053, respectively) and at 40% and 70% (*P = 0.0263 and 0.0473, respectively). TT, time trial; TTE, time to exhaustion; ${\dot{V}}_{O_{2}\max}$, maximal oxygen uptake test.

Mean normalised EMG responses during the ${\dot{V}}_{O_{2}\max}$, TT and TTE tests were 54.3 ± 13.9, 85.0 ± 9.2 and 71.9 ± 4.2, respectively. Normalised EMG differed by test (*P* < 0.001) and time (*P* < 0.001) (Figure 3). Pairwise comparisons showed that EMG was lower during ${\dot{V}}_{O_{2}\max}$ than during TT (*P* < 0.001) and TTE (*P* = 0.00100), and higher during TT than during TTE (*P* < 0.001). Across the time‐normalised analysis, TT was higher than ${\dot{V}}_{O_{2}\max}$ at 10%, 20%, 30%, 40% and 70% of test completion (*P* = 0.00540, *P* < 0.001, *P* = 0.00530, *P* = 0.0263 and *P* = 0.0473, respectively).

### 3.4 Near infrared spectroscopy

#### 3.4.1 Tissue oxygenation index

**FIGURE 4:**
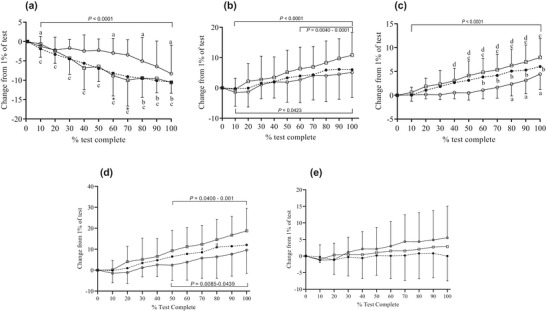
Changes TOI (a), OHb (b), HHb (c), tHb (d) and HBdiff (e) during the ${\dot{V}}_{O_{2}\max}$ (¡), TT (l) and TTE (o) tests. Values are means ± SD for 13 participants, with each participant contributing repeated measures across normalised % test‐completion bins. (a) TT and TTE vs. ${\dot{V}}_{O_{2}\max}$ at 60% and 70% (*P = 0.0308 and *P *= 0.0400, respectively). TOI in ${\dot{V}}_{O_{2}\max}$ decreased from 10% to 80% (*^a^P *= 0.0169, 0.0209, 0.0144, respectively). TT at 70%, 80%, 90% and 100% compared to 10% (*^b^P *= 0.0266, 0.0166 and 0.0159, respectively). TTE from 10% to 100% (*^c^P < *0.0001). (b) OHb over time (*P *< 0.0001). OHb increased 10% to 100% for ${\dot{V}}_{O_{2}\max}$ (*P *= 0.0423) and from 60% to 100% (*P *= 0.0040–0.0001) for TTE. (c) Significant effect for time (*P* < 0.0001). ${\dot{V}}_{O_{2}\max}$ HHb from baseline to 80% (*^a^P *= 0.0088), 90% (*P *= 0.0026) and 100% (*P *< 0.0001). TT HHb baseline from 60% (*^b^P *= 0.0105), 70% (*P *< 0.0001) and 80–100% (*P *< 0.0001). TTE HHb from 50% (*^c^P *= 0.0251–0.0010). TTE vs. ${\dot{V}}_{O_{2}\max}$ at 40%–90% (*^d^P *= 0.0078–0.0343). (d) ${\dot{V}}_{O_{2}\max}$ tHb (50% to 100%, *P *= 0.008–0.043), TT from 70% (*^a^P *= 0.0043) to 80%, (*P *= 0.0252). TTE from 50% to 100% (*P *= 0.0400–0.0012). TT, time trial; TTE, time to exhaustion; ${\dot{V}}_{O_{2}\max}$, maximal oxygen uptake test.

TOI decreased significantly across time (*P* < 0.0001, *n* = 13) with a test × time interaction (*P* < 0.0001) (Figure 4a). *Post hoc* analysis revealed TT and TTE to have decreased to a greater degree than ${\dot{V}}_{O_{2}\max}$ at 60% and 70% (−8.5 ± 5.9 vs. −2.9 ± 3.7 and −9.5 ± 6.6 vs. −3.4 ± 3.9, respectively; *P* = 0.0308 and *P *= 0.0400, respectively), after which the difference diminished and was no longer significant. TOI in the ${\dot{V}}_{O_{2}\max}$ test decreased from 10% (−1.2 ± 2.6), 60% (−2.9 ± 3.9) and 80% (−5.04 ± 6.0) to 100% (−8.3 ± 7.4) (*P* = 0.0169, 0.0209 and 0.0144, respectively). TT was significantly lower at 70%, 80%, 90% and 100% compared to 10% (−9.1 ± 6.6, −9.5 ± 6.6, −10.1 ± 6.8, −10.5 ± 7.2 and −1.9 ± 1.9, respectively; *P *= 0.0325, 0.0266, 0.0166 and 0.0159, respectively). TTE decreased from 10% (−0.65 ± 3.4) through to 100% (−10.6 ± 2.8; *P* = 0.0138–0.0001).

#### 3.4.2 Oxyhaemoglobin

There was a significant increase in OHb over time (*P* < 0.0001) (Figure 4b). *Post hoc* tests revealed that OHb increased significantly during ${\dot{V}}_{O_{2}\max}$ from 10% to 100% of test completion (*P *= 0.0423) and during TTE from 60% to 100% (*P* ranged from 0.0040 to 0.0001), whereas no differences were apparent for TT (*P* = 0.0915). There were no differences detected between tests.

#### 3.4.3 Deoxyhaemoglobin

For HHb, there a significant effect of time (*P* < 0.0001), with a significant effect of test (*P *= 0.0005). *Post hoc* comparisons indicated that, within the ${\dot{V}}_{O_{2}\max}$ test, HHb increased from baseline at 80% (*P *= 0.0088), and at 90% (*P *= 0.0026) and 100% (*P *= 0.0005). Within TT HHb differed from baseline at 60% (*P* = 0.0105) 70% (*P* = 0.0001) and 80–100% (*P* < 0.0001). Within TTE, HHb differed from baseline at 50% onwards (*P* = 0.0251–0.0010). Between‐test comparisons at each time point showed that TTE differed from ${\dot{V}}_{O_{2}\max}$ at 40–90% (*P* = 0.0343–0.0107), with no differences at 10% (*P* = 0.246) or 100% (*P* = 0.0974).

#### 3.4.4 Total haemoglobin and haemoglobin difference

Over the normalised time course, tHb exhibited trial‐specific increases (Figure 4d). In ${\dot{V}}_{O_{2}\max}$, tHb increased chiefly late in the test from 50% (*P* = 0.0439), 60% (*P* = 0.0085), 70% (*P* = 0.0405) and 80% (*P* = 0.0381), whereas in TT it rose earlier and remained elevated from 70% (*P* = 0.0043) and 80% (*P *= 0.0252). The TTE showed increases from 50% to 100% (*P* = 0.0400–0.0012). Hbdiff changes were smaller (Figure 4e), with no significant differences between tests although there was a main effect of time (*P* < 0.0001) but pairwise comparison tests between individual time points were not significant (*P* = 0.442) after correction for multiple comparison. This suggests that the time effect reflects a modest overall temporal pattern rather than a difference between specific time points, indicating that the dominant between‐trial difference over time was in perfusion (tHb) rather than a large shift in extraction where TOI trends were consistent with increasing late‐task desaturation.

### 3.5 Perceptual and emotional responses

#### 3.5.1 RPE

Rating of perceived exertion was not significantly (*P* = 0.687) different across trials. Average values for each test are shown in Table 1.

#### 3.5.2 Feeling scale

**FIGURE 5:**
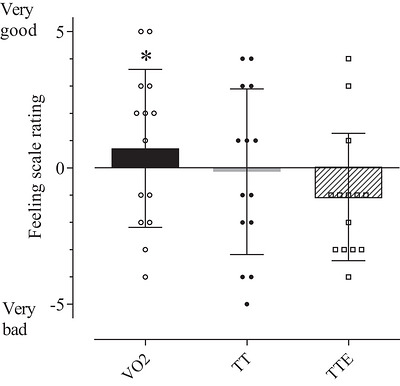
Feeling scale responses to each exercise model. FS was higher after the ${\dot{V}}_{O_{2}\max}$ test than TT and TTE (*P = 0.0412 and 0.0124, respectively). TT, time trial; TTE, time to exhaustion; ${\dot{V}}_{O_{2}\max}$, maximal oxygen uptake test. *n* = 14.

Normalised FS data showed that participants rated how they felt at the end of the ${\dot{V}}_{O_{2}\max}$ test as more pleasant (6.7 ± 2.9) than either the TT (5.9 ± 3.0) or the TTE (4.9 ± 2.3) (*P *= 0.0412 and 0.0124, respectively) (see Figure 5).

### 3.6 The end spurt

#### 3.6.1 Power output, EMG and NIRS responses

**FIGURE 6:**
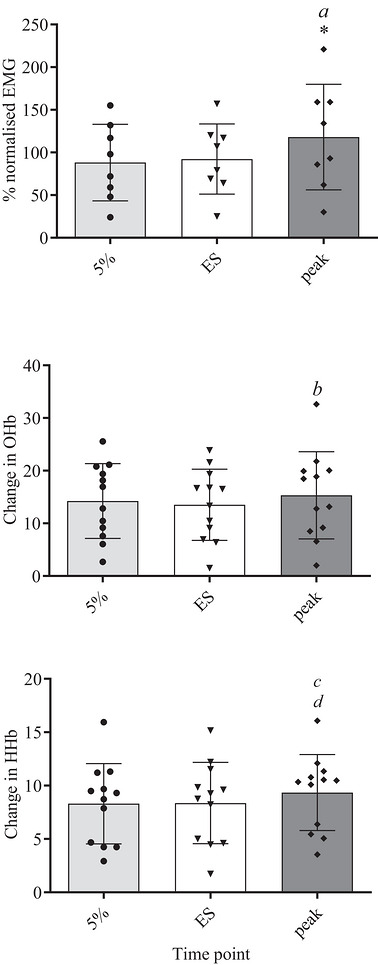
Characteristics of the electromyography (EMG) and near infrared spectroscopy (NIRS) of the prefrontal cortex for oxyhaemoglobin (OHb) and deoxyhaemoglobin (HHb) in response to the end spurt (ES) from 5% pre ES (5%), and at peak power attainment (peak). EMG peak vs. ES (*P = 0.0415) and 5% (*^a^P *= 0.0396). Peak OHb vs. ES (*^b^P *= 0.0462). HHb ES vs. peak (*^c^P *= 0.0046) and 5% to peak (*^d^P *= 0.0010).

PO increased from 313.0 ± 51.7 W at the start of the ES to a peak PO of 384.2 ± 71.3 W (*P *= 0.00910). The ES was characterised by significantly higher normalised EMG activity at peak power attainment (118 ± 61.8%) compared to the start of the ES (92.2 ± 41.1, *P *= 0.0415) and at 5% (*P* = 0.0396) pre ES (88.1 ± 45.0) (Figure 6). There was an increase in absolute values of OHb (13.3 ± 7.3 to 15.2 ± 8.6, *P *= 0.0462) from ES to peak and similarly for HHb from ES to peak (8.3 ± 3.8 to 9.3 ± 3.6, *P *= 0.0046) and from 5% to peak (8.4 ± 3.7 to 9.3 ± 3.6, *P *= 0.0010) (Figure 6).

## 4 DISCUSSION

The aims of this study were to examine the neurophysiological response to three different exercise models, the ${\dot{V}}_{O_{2}\max}$ test, a TT and a TTE test, and to evaluate and describe the differences in cerebral responses to self‐regulated exercise via the ES. As hypothesised, the novel findings were that each exercise model showed different cerebral characteristics and that the ES preceded an increase in PO and EMG activity which coincided with greater deoxygenation at the PFC. This adds weight to the notion that different exercise models for measuring endurance are underpinned by different neurophysiological processes. To our knowledge, these comparative findings have yet to be reported.

### 4.1 Neurophysiological responses during exercise

During exercise, cerebral oxygenation reflects the balance between O_2_ delivery and utilisation. In our data, TOI declined progressively, indicating net desaturation across tests, while concurrent rises in OHb and HHb are best interpreted using combined indices of total haemoglobin (tHb = OHb + HHb) as a gauge of regional blood volume and perfusion, with haemoglobin difference (Hbdiff = OHb − HHb) together with TOI to index the delivery–utilisation balance, rather than from OHb or HHb in isolation (Figure 4). These trajectories suggest that the rises in cerebral perfusion depend on the exercise paradigm: self‐paced work (TT) accrues volume earlier, incremental loading (${\dot{V}}_{O_{2}\max}$) shows a late hyperaemic push near failure, and constant work (TTE) largely blunts that rise. Because ΔHb changed less than tHb, we attribute the main temporal divergence to volume and perfusion differences, with extraction and desaturation (TOI/Hbdiff) changing more modestly across the 10% bins.

In keeping with prior work, increases in tHb (with parallel OHb and HHb rises) are consistent with increased regional blood volume during exercise up to high intensities; beyond ventilatory compensation, cerebral oxygenation can decline with a short delay (Bhambhani et al., 2007; Billaut et al., 2010). Earlier studies also reported TOI declines or plateaus as intensity becomes severe and prolonged (González‐Alonso et al., 2004). Crucially, we do not infer perfusion or extraction from OHb or HHb alone: tissue O_2_ extraction is interpreted from ΔHb in conjunction with TOI, while putative flow and volume changes are attributed to tHb (or nTHI where available), not to OHb per se (Figure 4) (Zauner & Muizelaar, 1997).

Comparing paradigms, TT and TTE exhibited larger TOI reductions by mid‐test (50–70%) than ${\dot{V}}_{O_{2}\max}$; the subsequent TOI decline in ${\dot{V}}_{O_{2}\max}$ narrowed these differences so that end‐exercise saturation was similarly reduced across tests. OHb increased over time in all paradigms; where TTE showed greater OHb late (80–100%) when accompanied by a rise in tHb is consistent with higher cerebral blood volume and perfusion, whereas, Hbdiff remained comparatively stable, indicating only modest shifts in extraction relative to delivery. Together, these patterns imply paradigm‐specific neural–vascular control at the PFC, with protocol structure shaping when volume builds and how strongly oxygenation balance shifts toward extraction.

A further implication of these findings is that the exercise models appeared to differ more in the trajectory by which cerebral oxygenation/perfusion changed than in the final oxygenation state attained at exercise termination. TT and TTE exhibited larger reductions in TOI by mid‐test, whereas the subsequent decline in ${\dot{V}}_{O_{2}\max}$ narrowed these differences so that all three paradigms converged toward a similarly reduced end‐exercise oxygenation state (Figure 4a). At the same time, the pattern of tHb suggests that the tests differed as to when cerebral perfusion was instigated, with a later rise during ${\dot{V}}_{O_{2}\max}$ and earlier or more sustained increases during TT and TTE (Figure 4d), while Hbdiff changed comparatively little (Figure 4e). Thus, protocol structure appears to alter the route to exhaustion, rather than impose a wholly different terminal cerebral constraint.

Finally, our interpretation is consistent with a recent systematic review concluding that muscle and brain show distinct oxygenation responses during exercise, with skeletal muscle tending toward greater O_2_ extraction, the brain toward maintaining or increasing O_2_ delivery, with both systems ultimately constrained near exhaustion (Orcioli‐Silva et al., 2024).

As might be expected, EMG responses followed the PO patterns of each test. The dynamic tracking of EMG to work rate has been previously proposed and suggested to be related to central regulation of exercise intensity (St Clair Gibson et al., 2001; Billaut et al., 2010). The ${\dot{V}}_{O_{2}\max}$ test was most different in PO compared to the TT and TTE, as would be expected. The TT, while being the same average PO as the TTE for each participant, was lower than TTE at the start (1%), in the middle (60% and 70%) and then higher at the end (90% and 100%). Thus, despite on average having the same mean PO, it is possible to observe that the regulatory nature of the TT allowed for shifts in PO during the TT to adjust for the physiological strain imposed by that intensity. Notably, despite TTE being the same average PO as the TT, the TTE continued for a significantly longer period than the TT (∼138 s). Therefore, the fluctuations in the PO of the TT allow for a maximal effort to be completed, but without the fluctuations in power, the effort can be sustained for a much longer period.

### 4.2 Neurophysiological responses to the end of exercise

In changes at the end of exercise compared to the first percent, NIRS showed the TT to have a greater increase in HHb than either ${\dot{V}}_{O_{2}\max}$ or TTE. This was related to the ES. Up‐regulation of cerebral responses was aligned with the ES, in that increases in OHb and HHb occurred concurrently with increases in PO and EMG response. These differences between TT and ${\dot{V}}_{O_{2}\max}$ tests have been shown previously with NIRS measures at the musculature. Previously, others (Neary et al., 2002) have presented evidence suggesting that a maximal effort in a ${\dot{V}}_{O_{2}\max}$ test and a TT event involve different physiological mechanisms. Lower muscle oxygenation levels were found during a TT event compared to exhaustion in a ${\dot{V}}_{O_{2}\max}$ test, suggesting that during a ${\dot{V}}_{O_{2}\max}$ test a limit of oxygen delivery nor extraction is the case as the body can tolerate a greater degree of desaturation when able to regulate exercise. Further to this, the ability to tolerate higher muscle de‐oxygenation levels has been found to be linked to performance in a TT (Neary et al., 2005). This may be the case for cerebral de‐oxygenation; there is a higher degree of cerebral de‐oxygenation from the ES compared to that obtained at the end of the ${\dot{V}}_{O_{2}\max}$ test, suggesting the importance of intensity regulation in achieving higher levels of cerebral desaturation. Greater de‐oxygenation has been seen in line with increases in PO in a known versus unknown TT distance performance (Wingfield et al., 2018). This suggests that cerebral changes may relate to the neural regulation of exercise, and de‐oxygenation may linked to exercise termination. The ability to defend against such decreases during self‐paced exercise has been linked to endurance performance (Santos‐Concejero et al., 2017).

The end‐spurt data further suggest that a key feature of self‐paced exercise is not simply a higher final PO, but the capacity to defer a greater degree of cerebral and neuromuscular strain until the remaining exercise duration is sufficiently short. In the TT, PO was redistributed across the trial, being lower earlier and higher toward the end compared with the other paradigms (Figure 2), while EMG tracked this pattern and was greater in TT than ${\dot{V}}_{O_{2}\max}$ at several points across exercise (Figure 3). More specifically, during the ES itself, the late rise in PO was accompanied by increased EMG and concurrent increases in OHb and HHb (Figure 6), indicating that the ES reflects a coordinated escalation in motor output and cerebral oxygenation strain rather than a purely mechanical rise in work rate. This interpretation is strengthened by the observation that TT and TTE were matched closely in average power, yet TT was completed in less time (Table 1), implying that self‐paced regulation allows a higher‐intensity late effort to be expressed that is not sustained during constant‐load exercise.

A key differentiator across the three tests may be the psychological and physical strain induced, especially towards exercise termination. While RPE has been proposed to be an integrator of both these strains (Tucker, 2009), there are data that suggest they are separable and distinct (Swart et al., 2009). Swart et al. (2009) concluded that the decision to terminate exercise may be a balance between motivation and affect and an overall sense of effort. Perceptual responses to exercise have also been theorised to make a person feel increasingly bad and require cognitive processes to avoid slowing down or stopping the task (Behrens et al., 2023). This may be the case with the ${\dot{V}}_{O_{2}\max}$ test and TTE: a TTE being constant load may have lower afferent feedback compared to an increasing requirement for power in the ${\dot{V}}_{O_{2}\max}$ test, and the psychological demand of maintaining a constant PO (TTE) compared to a continually increasing PO may be different.

Notably, there is a dissociation between effort and affect at exercise termination. Despite there being no between trial differences in RPE (Table 1), participants rated the ${\dot{V}}_{O_{2}\max}$ test as more pleasant than either TT or TTE (Figure 5; Table 1). This suggests that the exercise models were of similar effort at the point of termination, but not similarly experienced. One possible interpretation is that the paradigm influences the affective cost of sustaining or increasing intensity, even when the conscious sense of exertion is comparable. Within this framework, the self‐paced TT may allow participants to regulate work rate in a way that preserves task completion, but at the expense of greater unpleasantness late in the task and a greater tolerance of cerebral deoxygenation during the ES.

### 4.3 Limitations

To make these tests comparable, a workload and average PO approach was taken. However, the nature of the tests dictates a variation in intensity. Data from this study were not computed in line with individual respiratory compensation points (RCP). The RCP is an intensity at which known changes in neurophysiology occur (Bhambhani et al., 2007; Robertson & Marino, 2015), and therefore the intensity of the tests relative to the RCP may have impacted individual cerebral responses.

A limitation to consider is that our participants were aerobically trained, which improves economy and alters ventilatory and cerebrovascular regulation. As such, the size and timing of NIRS changes observed in the present study may differ in untrained individuals. Untrained cohorts often reach comparable relative intensities at lower absolute workloads and may demonstrate steeper HHb/TOI changes and earlier cortical desaturation. Accordingly, our findings should be interpreted as representative of trained adults with replication in untrained and more diverse samples (age, sex, training background) warranted. It is also important to note that referencing NIRS to rest can exaggerate transition‐related offsets and between‐visit baseline drift. We, therefore, anchored signals to the earliest stable on‐task epoch (1%); although initial workload differed slightly by paradigm, this choice improves within‐context comparability and does not alter our trajectory‐based inferences.

### 4.4 Conclusion

In summary, the three exercise models produced distinct temporal patterns of prefrontal oxygenation, perfusion and muscle activation. TT and TTE showed earlier declines in TOI than ${\dot{V}}_{O_{2}\max}$, but all three paradigms converged to a similarly reduced end‐exercise brain oxygenation. The self‐paced TT additionally displayed a characteristic end spurt with increased PO and EMG, whereas ${\dot{V}}_{O_{2}\max}$ was experienced as more pleasant than TT and TTE. These findings suggest that exercise protocol structure influences the route to exhaustion more than the final cerebral oxygenation state.

## AUTHOR CONTRIBUTIONS

Caroline V. Robertson: Investigation, methodology, design, data collection and analyses, writing – original draft. Nicole T. Vargas: Investigation, data collection, writing & review/editing. Tegan Hartmann: Investigation, writing review/editing. Frank E. Marino: Investigation, supervision, writing, review/editing, resources. All authors have read and approved the final version of this manuscript and agree to be accountable for all aspects of the work in ensuring that questions related to the accuracy or integrity of any part of the work are appropriately investigated and resolved. All persons designated as authors qualify for authorship, and all those who qualify for authorship are listed.

## CONFLICT OF INTEREST

None declared.

## Data Availability

All data supporting the results are provided in the manuscript.
